# Verifying the Relative Efficacy between Continuous Positive Airway Pressure Therapy and Its Alternatives for Obstructive Sleep Apnea: A Network Meta-analysis

**DOI:** 10.3389/fneur.2017.00289

**Published:** 2017-06-28

**Authors:** Tingwei Liu, Wenyang Li, Hui Zhou, Zanfeng Wang

**Affiliations:** ^1^Department of Respiratory Medicine, First Affiliated Hospital of China Medical University, Shenyang, China

**Keywords:** obstructive sleep apnea, Apnea–Hypopnea Index, Epworth Sleepiness Scale, blood pressures, network meta-analysis

## Abstract

Obstructive sleep apnea (OSA) is a common breathing disorder, and continuous positive airway pressure (CPAP) therapy together with its alternatives has been developed to treat this disease. This network meta-analysis (NMA) was aimed to compare the efficacy of treatments for OSA. Cochrane Library, MEDLINE, and Embase were searched for eligible studies. A conventional and NMA was carried out to compare all therapies. Sleeping characteristics, including Apnea–Hypopnea Index (AHI), Epworth Sleepiness Scale (ESS), arterial oxygen saturation, and arousal index (AI), and changes of blood pressure were selected as outcomes. A total of 84 studies were finally included after rigorous screenings. For the primary outcomes of AHI and ESS, the value of auto-adjusting positive airway pressure (APAP), CPAP, and oral appliance (OA) all showed statistically reduction compared with inactive control (IC). Similar observation was obtained in AI, with treatments of the three active interventions. A lower effect of IC in SaO_2_ was exhibited when compared with APAP, CPAP, and OA. Similar statistically significant results were presented in 24 h systolic blood pressure and 24 h DBP when comparing with CPAP. Our NMA identified CPAP as the most efficacious treatment for OSA patients after the evaluation of sleeping characteristics and blood pressures. In addition, more clinical trials are needed for further investigation due to the existence of inconsistency observed in this study.

## Introduction

Obstructive sleep apnea (OSA) is a common breathing disorder which is identified by repetitive air flow reduction or cessation during sleep (1). The prevalence of OSA is estimated between 2 and 4%, varying with obesity status, gender, and age of populations (2, 3), usually caused by repetitive pharynx dysfunction which leads to apnea and hypopnea that result in the down regulation of blood oxygen levels (4). Oxygen desaturation triggered by chronic hypoxia further causes repetitive arousals and significant changes in both transmural and intra-thoracic pressure. This mechanism can increase the sympathetic activity and oxidative stress on the heart and intra-thoracic vessels, eventually resulting in vascular damages (5).

The severity of OSA can be classified by the Apnea–Hypopnea Index (AHI), evaluating the episode frequency of apnea/hypopnea in 1 h (4), which can be used to predict the relative risk of OSA. For instance, a 10% weight loss is predicted to be correlated with a 26% decrease in the AHI (6). OSA can also be evaluated by using the Epworth Sleepiness Scale (ESS), which considers both daytime sleepiness and the average sleep propensity (2, 3).

Continuous positive airway pressure (CPAP) therapy is a highly effective treatment option for OSA patients (7). Massive evidence suggests that CPAP therapy not only improves the AHI of OSA patients but also stabilizes their blood pressure levels (8, 9). Besides that, CPAP therapy is able to provide OSA patients with additional reduction in both systolic blood pressure (SBP) and diastolic blood pressure (DBP) (2, 3). One major challenge of CPAP therapy is to identify a specifically effective pressure for individual OSA patient before CPAP therapy can be continuously applied to patients. This is usually achieved through standard manual titration and it is a time-consuming task (10). The above issue may be overcome by the auto-adjusting positive airway pressure (APAP) that only applies the lowest effective pressure to patients, and the corresponding pressure delivered is continuously adjusted depending on the residual symptoms detected on patients (11). CPAP also causes discomfort or nasal problems, and thus, it is not tolerable for all OSA patients (12). As a result, oral appliance (OA) therapy has been developed as an alternative to CPAP therapy for preventing airway collapse. APAP was reported to have equivalent performance in improving sleepiness compared to CPAP therapy (13). Although OA therapy is able to improve the AHI in OSA patients, several indexes of the OA therapy are inferior to those of CPAP therapy (14). Moreover, using mandibular advancement devices (an OA therapy) was associated with a reduction in SBP and DBP among OSA patients. However, such a benefit was not observed in OSA patients with CPAP therapy (2, 3).

Evidence in the current literature mainly comes from meta-analysis which was designed to answer the above questions. However, some conflicting results and conclusions appeared to be a major issue and this may arise from variations in study design, size, and participants (6, 15, 16). Thus, a network meta-analysis (NMA) with a large scale should be designed to integrate current MAs and clinical trials, increase the level of evidence and the credibility of individual studies as well as to provide clinicians with genuine consensus for the purpose of compensating the lack of head-to-head comparison.

## Materials and Methods

### Identification of Trials

We comprehensively investigated the databases of the Cochrane Library, MEDLINE, and Embase. Key words and subject terms included “obstructive sleep apnea,” “physical therapy modality,” “continuous positive airway pressure,” “auto-adjusting positive airway pressure,” and “oral appliance.” Controlled trials were identified using the Cochrane Highly Sensitive Search Strategy (sensitivity-maximizing and precision-maximizing version). The references of any related records were also screened in order to include additional qualified trials. Moreover, all articles screened and reviewed were published in English.

### Inclusion Criteria

Trials were supposed to meet the following standards: (1) controlled trials were preferred in our study selection. Other types of studies were also included if their research topics are relevant; (2) trials or studies must recruit patients who were older than 18 years and diagnosed with OSA. (3) OSA was specifically defined as AHI > 5/h; (4) At least two of the following treatments were compared: CPAP, APAP, OA, and inactive control (IC, such as sham CPAP and placebo). (4) The outcomes of each study should include at least one of sleeping characteristics or blood pressure, including AHI, ESS, arterial oxygen saturation (SaO_2_), arousal index (AI), SBP, and DBP. AHI and ESS were assessed as primary outcomes, while the other outcomes were used as secondary outcomes.

### Data Extraction

We recorded basic characteristics of trials, including information of publications (author, year, and country), design of trials (RCT or non-RCT, and blinding), and follow-up durations. The changes of indexed, which were related to the quality of sleep and blood pressure, were seen as the most significant part of the trials. All the endpoints were continuous valuables, so the weighted mean difference (WMD) considering the trial size between different therapies was computed as well as corresponding sample SD.

### Statistical Methods

STATA 12.0 software (Stata Corp, College Station, TX, USA) was used to perform traditional MA and WMD with corresponding 95% confidence interval (CI) were computed. We assessed heterogeneity among the included studies by Cochran’s *Q* test (17) and the *I*^2^ test (18). If *P* > 0.1 and *I*^2^ < 50%, it suggested no significant heterogeneity existed and fixed-effects model was used. Otherwise, the random-effects model was applied if there was significant heterogeneity.

We combined direct and indirect evidence by NMA, a Bayesian framework based on Markov chain Monte Carlo method. STATA 12.0 software (Stata Corp, College Station, TX, USA) and WinBUGS software (MRC Bio-statistics Unit, Cambridge, UK) were applied as computational tools, which presented WMD with the corresponding 95% credible intervals (CrIs). In order to rank these therapies with respect to each clinical outcome, the surface of cumulative ranking curve area (SUCRA) was presented and generated a simulated ranking based on SUCRA values. For each comparison, the “design-by-treatment” interaction model was used to evaluate consistency between direct and indirect evidence. In the presence of significant inconsistency, the *P*-value of the “design-by-treatment” interaction model would be less than 0.05 and the result was displayed graphically in the node-splitting plots and net heat plots.

### Risk of Bias Assessment

We assessed the risk of bias by using the Cochrane Collaboration’s criteria. Each study was assessed with respect to several types of bias (performance, detection, selection, attrition, and reporting bias) and classified as being at low, unclear, or high risk of bias for each potential source of bias. A “comparison adjusted” funnel plot was exhibited in order to illustrate publication bias, and the degree of symmetry in the funnel plot indicated whether the small-study effect was significant or not.

## Results

### Characteristics of Trials and Patients

**Figure 1:**
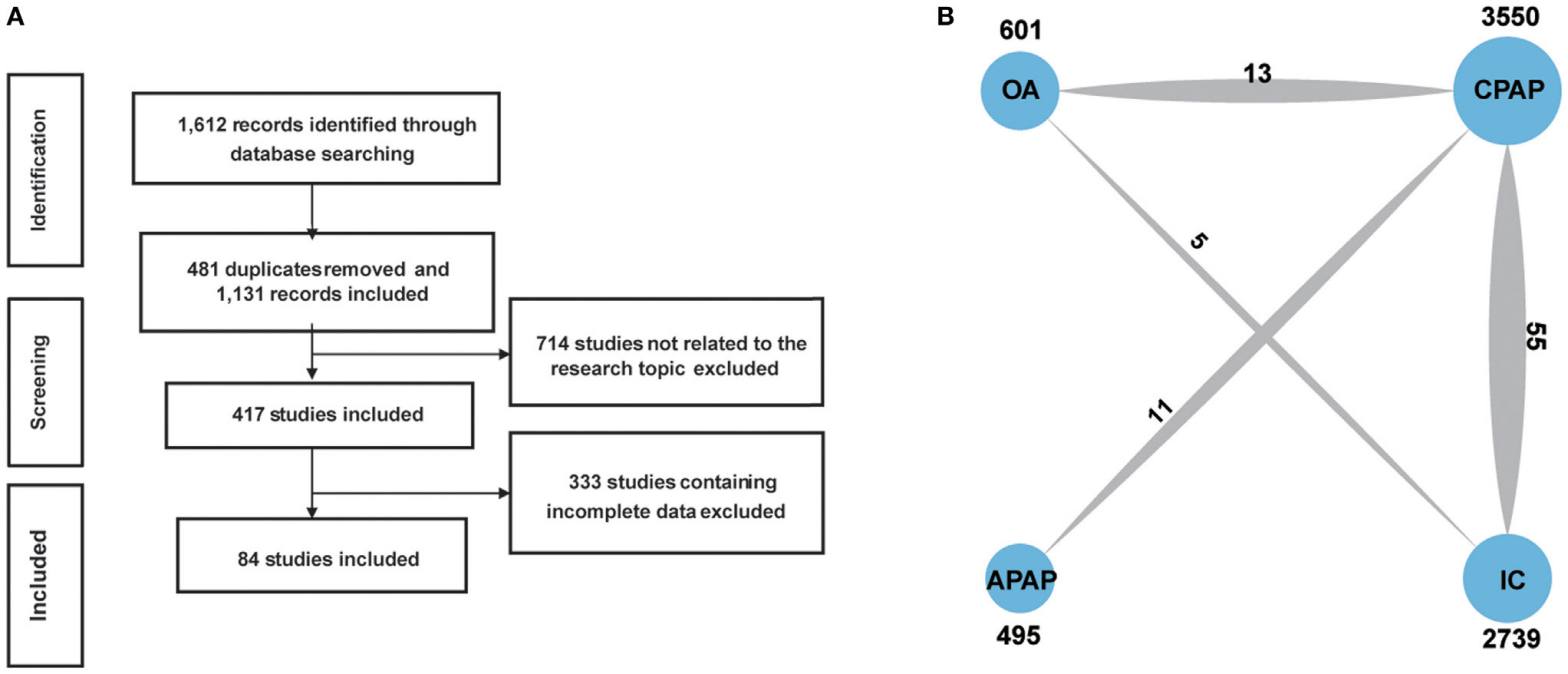
Flowchart **(A)** and network plot **(B)**. The network plot show direct comparison of different treatments, with node size corresponding to the sample size. The number of included studies for specific direct comparison decides the thickness of solid lines. Abbreviations: CPAP, continuous positive airway pressure; APAP, auto-adjusting positive airway pressure; IC, inactive control; OA, oral appliance.

We retrieved and screened literature in the process showed in Figure 1A. A total of 1,612 records were identified through database searching and 481 were removed as duplicates. We excluded 714 records by reviewing their topics or abstracts. Another 333 records were removed after full-text reading since they contained incomplete data. A total of 84 studies were finally included and RCTs (9, 11, 13, 19–99). The pattern of evidence provided by studies was displayed in the network plot (Figure 1B). The size of nodes represents the sample size, and the thickness of lines indicates the number of trials comparing two therapies. CPAP was investigated in most trials. The baseline characteristics of studies were recorded in Table 1. Trials collected in our study were conducted around the world, 41 in Europe, 20 in North America, 6 in Brazil, 5 in China, 8 in Australia, and 1 in New Zealand, Japan, India, and Pakistan, respectively.

**Table 1:** Main characteristics of included studies.

Reference	Country	Design	Blinding	Follow-up	Treatment 1							Treatment 2						
					t1	*n*	Age	Male (%)	BMI	AHI (h^−1^)	ESS	t2	*n*	Age	Male (%)	BMI	AHI (h^−1^)	ESS
Salord et al. (19)	Spain	RCT	Double	–	CPAP	42	48.5 ± 8.6	26	45.7 ± 5	68.3 ± 11	7.9 ± 4.5	IC	38	44.6 ± 9.4	29	45.7 ± 5	68.3 ± 11	7.9 ± 4.5
Pepin et al. (11)	France	RCT	Double	4 months	CPAP	133	58 ± 3.5	70.8		37.7 ± 6.5	9 ± 2	APAP	143	58 ± 3.2	69.6	–	37.7 ± 6.5	9 ± 2
Paz et al. (20)	USA	RCT	Double	1 months	IC	74	51.0 ± 11.7	52.7	37.3 ± 8.3	20.6 ± 6.2	10 ± 1.2	CPAP	75	51.7 ± 11.8	54.7	37.3 ± 8.3	20.6 ± 6.2	10 ± 1.2
Pamidi et al. (21)	Canada	RCT	Open-label	2 months	CPAP	26	53.8 ± 6.2	62	36.8 ± 7.8	34.2 ± 24.5	10 ± 5.9	OA	13	55.2 ± 8.4	77	36.8 ± 7.8	34.2 ± 24.5	10 ± 5.9
Muxfeldt et al. (22)	Brazil	RCT	Open-label	6 months	IC	60	60.2 ± 8.4	41.7	33.8 ± 5.8	39 ± 18	12 ± 6	CPAP	57	60.8 ± 8.0	37.9	33.8 ± 5.8	39 ± 18	12 ± 6
Martinez-Garcia et al. (23)	Spain	RCT	Open-label	–	CPAP	115	75.5 ± 3.8	63.5	33 ± 7.3	53.5 ± 15.6	9.6 ± 4	IC	109	75.6 ± 4.0	73.4	33 ± 7.3	53.5 ± 15.6	9.6 ± 4
Huang et al. (24)	China	RCT	Single	6 months	IC	37	62.7 ± 6.7	86.5	27.5 ± 2.6	28.7 ± 12.4	8.3 ± 3.4	CPAP	36	62.0 ± 6.8	77.8	27.5 ± 2.6	28.7 ± 12.4	8.3 ± 3.4
Dalmases et al. (25)	Spain	RCT	Open-label	3 months	CPAP	17	70.8 ± 5.1	64.7	32.8 ± 3.9	61.2 ± 17.9	7.94 ± 2.99	IC	16	71.9 ± 6.0	75	32.8 ± 3.9	61.16 ± 17.86	7.94 ± 2.99
Woodson et al. (26)	USA	RCT	Open-label	12 months	CPAP	23	57.1 ± 10	95.6	28.4 ± 2.4	31.3 ± 12.3	11.2 ± 5.3	IC	23	52.7 ± 10.4	82.6	28.4 ± 2.4	31.3 ± 12.3	11.2 ± 5.3
Neikrug et al. (27)	USA	RCT	Open-label	6 weeks	CPAP	19	66.7 ± 8.5	63.2	27.8 ± 4.7	21.1 ± 14.9	–	IC	19	67.7 ± 10.0	73.7	27.8 ± 4.7	21.1 ± 14.9	–
Lloberes et al. (28)	Spain	RCT	–	–	CPAP	36	58.3 ± 9.4	70.7	31.4 ± 4.9	50.1 ± 21.6	6.76 ± 3.7	IC	42	58.3 ± 9.4	70.7	31.4 ± 4.9	50.1 ± 21.6	6.76 ± 3.7
Gottlieb et al. (29)	USA	RCT	–	3 months	CPAP	90	63.5 ± 7.0	76	33 ± 5	25.4 ± 8.7	8 ± 3.8	IC	97	63.1 ± 7.7	78	33 ± 5	25.4 ± 8.7	8 ± 3.8
Chasens et al. (30)	USA	RCT	Double	1 month	CPAP	12	–	58		50.2 ± 30.9	11.42 ± 4.62	IC	11	–	64	–	50.2 ± 30.9	11.42 ± 4.62
Berry and Sriram (13)	USA	RCT	–	–	APAP	78	57.7 ± 12.1	93.5	34.2 ± 5.8	–	15.2 ± 4.4	CPAP	70	59.7 ± 12.6	92.3	34.2 ± 5.8	–	15.2 ± 4.4
Schutz et al. (31)	Brazil	RCT	–	2 months	CPAP	9	38.62 ± 8.15	–	25.9 ± 5.31	25.1 ± 10.5	–	OA	9	42.33 ± 6.20	–	25.9 ± 5.31	25.1 ± 10.5	–
Phillips et al. (32)	Australia	RCT	Open-label	1 month	CPAP	56	49.5 ± 11.2	80.9	29.5 ± 5.5	25.6 ± 12.3	9.1 ± 4.2	OA	52	49.5 ± 11.2	80.9	29.5 ± 5.5	25.6 ± 12.3	9.1 ± 4.2
Pedrosa et al. (33)	Brazil	RCT	Open-label	6 months	CPAP	19	57.0 ± 2.1	74	36 ± 2.5	36 ± 6.5	12 ± 4	IC	16	55 ± 2	81	36 ± 2.5	36 ± 6.5	12 ± 4
Martinez-Garcia et al. (9)	Spain	RCT	Single	3 months	CPAP	98	57.8 ± 9.5	72.4	34.3 ± 5.7	41.3 ± 18.7	8.9 ± 4	IC	96	58.2 ± 9.6	64.6	34.3 ± 5.7	41.3 ± 18.7	8.9 ± 4
Diaferia et al. (34)	Brazil	RCT	–	3 months	IC	24	48.1 ± 11.2	100	27.4 ± 4.9	27.8 ± 20.3	12.7 ± 3	CPAP	27	48.1 ± 11.2	100	27.4 ± 4.9	27.8 ± 20.3	12.7 ± 3
Andren et al. (35)	Sweden	RCT	Single	3 months	OA	36	57.0 ± 8.0	83	30 ± 4	23 ± 16	11 ± 5.4	IC	36	59 ± 9	75	30 ± 4	23 ± 16	11 ± 5.4
Sivam et al. (36)	USA	RCT	Single	2 months	CPAP	27	47.0 ± 13.0	96.3	31.3 ± 3.8	37.2 ± 24.7	10 ± 4.8	IC	27	47 ± 13	96.3	31.3 ± 3.8	37.2 ± 24.7	10 ± 4.8
Lee et al. (37)	USA	RCT	Double	1 month	CPAP	26	48.3 ± 9.3	84.6	29.8 ± 4.6	36.7 ± 21.8	–	IC	30	48.2 ± 9.0	83.3	29.8 ± 4.6	36.7 ± 21.8	–
Kushida et al. (38)	USA	RCT	Double	6 months	CPAP	453	52.2 ± 12.2	65.3	32.4 ± 7.3	39.7 ± 24.9	10.07 ± 4.26	IC	422	50.8 ± 12.2	65.7	32.4 ± 7.3	39.7 ± 24.9	10.07 ± 4.26
Hoyos et al. (39)	Australia	RCT	Double	3 months	CPAP	34	51.0 ± 12.3	–	31.6 ± 5.3	38.5 ± 14.7	10 ± 4	IC	31	46.4 ± 10.4	–	31.6 ± 5.3	38.5 ± 14.7	10 ± 4
Sharma et al. (40)	India	RCT	Double	3 months	CPAP	86	45.1 ± 8.0	84	33.8 ± 4.7	47.9 ± 19.6	14.8 ± 3.7	IC	86	45 ± 8	95	33.8 ± 4.7	47.9 ± 19.6	14.8 ± 3.7
Ryan et al. (41)	Canada	RCT	Open-label	6 months	IC	22	60.7 ± 10.3	86.4	27.3 ± 5.8	33.3 ± 16.4	4.5 ± 2.1	CPAP	22	62.8 ± 12.8	72.7	27.3 ± 5.8	33.3 ± 16.4	4.5 ± 2.1
Phillips et al. (42)	Australia	RCT	Single	2 months	CPAP	37	49.4 ± 13.2	92.1	32.1 ± 4.3	41.2 ± 23.9	11.2 ± 4.9	IC	37	49 ± 13	92.1	32.1 ± 4.3	41.2 ± 23.9	11.2 ± 4.9
Kohler et al. (43)	Swiss	RCT	Single	1 month	CPAP	20	63.6 ± 5.1	95	32.9 ± 6.5	36 ± 17.3	13.8 ± 2.6	IC	21	61.8 ± 7.5	100	32.9 ± 6.5	36 ± 17.3	13.8 ± 2.6
Drager et al. (44)	Brazil	RCT	–	3 months	IC	18	44.1 ± 7.0	–	29 ± 2.6	58 ± 23	11 ± 5	CPAP	18	43 ± 7	–	29 ± 2.6	58 ± 23	11 ± 5
Aarab et al. (45)	Netherlands	RCT	Single	12 months	OA	21	50.4 ± 8.9	80.9	27.1 ± 3.1	21.4 ± 11	–	CPAP	22	54.9 ± 10.1	68.2	27.1 ± 3.1	21.4 ± 11	–
Nguyen et al. (46)	USA	RCT	Double	3 months	CPAP	10	52.9 ± 11.6	80	30.1 ± 4.7	38.8 ± 21.4	–	IC	10	53.9 ± 10.8	100	30.1 ± 4.7	38.8 ± 21.38	–
Lozano et al. (47)	Spain	RCT		3 months	IC	35	59.2 ± 10.9	62.9	31.5 ± 5.6	46.8 ± 21.4	–	CPAP	29	59.2 ± 8.7	75.9	31.5 ± 5.6	46.78 ± 21.43	–
Lam et al. (48)	China	RCT	Double	1 week	CPAP	31	46.5 ± 10.8	27.8 ± 3.7	27.8 ± 3.8	33.4 ± 9.5	10.3 ± 4.9	IC	30	46.5 ± 10.8	27.2 ± 3.7	27.8 ± 3.8	33.4 ± 9.5	10.3 ± 4.9
Durán-Cantolla et al. (49)	Spain	RCT	Double	12 weeks	CPAP	169	53.2 ± 10.2	79	31.9 ± 5.7	39.8 ± 22.7	10.3 ± 4.2	IC	171	51.7 ± 10.8	84	31.9 ± 5.7	39.8 ± 22.7	10.3 ± 4.2
Barbe et al. (50)	Spain	RCT	Open-label	12 months	CPAP	179	55 ± 10	82	32 ± 5	43 ± 19	6.4 ± 2.4	IC	177	56 ± 10	85	32 ± 5	43 ± 19	6.4 ± 2.4
Galetke et al. (51)	Germany	RCT	–	6 weeks	CPAP	19	51.7 ± 10.4	94.7	32.1 ± 5.7	40.5 ± 21.5	11.3 ± 4.7	APAP	15	52.1 ± 9.2	86.7	32.1 ± 5.7	40.5 ± 21.5	11.3 ± 4.7
Gagnadoux et al. (52)	France	RCT	–	2 months	CPAP	59	50.3 ± 9.1	–	26.7 ± 3.5	34.2 ± 13	10.6 ± 4.5	OA	59	50.3 ± 9.1	–	26.7 ± 3.5	34.2 ± 13	10.6 ± 4.5
Damjanovic et al. (53)	Germany	RCT	–	9 months	APAP	50	57.6 ± 2.1	74	31.8 ± 8.5	41.8 ± 24.7	8.5 ± 5.6	CPAP	50	59.4 ± 2.1	82	31.8 ± 8.5	41.8 ± 24.7	8.5 ± 5.6
Siccoli et al. (54)	UK	RCT	–	1 month	IC	51	48.7 ± 10.6	–	34.5 ± 5	–	15.2 ± 4	CPAP	51	48.1 ± 9.5	–	34.5 ± 5	–	15.2 ± 4
Ruttanaumpawan et al. (55)	Canada	RCT	–	1 month	IC	14	60.5 ± 10.3	85.7	32.3 ± 8.6	51.3 ± 15.6	–	CPAP	19	59.0 ± 7.8	94.7	32.3 ± 8.6	51.3 ± 15.6	–
Petri et al. (56)	Denmark	RCT	Double	1 month	OA	27	50 ± 11	85.2	30.7 ± 5.2	39.1 ± 23.8	11.7 ± 4.3	IC	29	49 ± 10	79.3	30.7 ± 5.2	39.1 ± 23.8	11.7 ± 4.3
Kohler et al. (57)	UK	RCT	Double	1 month	IC	49	48.7 ± 10.6	–	34.5 ± 5	–	15.2 ± 4	CPAP	50	48.1 ± 9.5	–	34.5 ± 5	–	15.2 ± 4
Hoekema et al. (58)	Neherlands	RCT	Open-label	3 months	OA	47	–	–	–	39.4 ± 30.8	–	CPAP	47	–	–	–	39.4 ± 30.8	–
Galetke et al. (59)	Germany	RCT	Single	2 months	CPAP	20	–	–	–	32.9 ± 19.1	10.3 ± 5.7	APAP	20	–	–	–	32.9 ± 19.1	10.3 ± 5.7
Egea et al. (60)	Spain	RCT	–	3 months	CPAP	28	64 ± 0.9	96	31.7 ± 12.7	43 ± 23.3	8 ± 3.7	IC	32	63 ± 1.6	91	31.7 ± 12.7	43 ± 23.3	8 ± 3.7
Cross et al. (61)	UK	RCT	Double	6 weeks	IC	29	48 ± 2	95.6	37 ± 5.4	63 ± 26.9	–	CPAP	29	–	–	37 ± 5.4	63 ± 26.9	–
West et al. (62)	UK	RCT	Double	3 months	CPAP	19	57.8 ± 10.4		36.6 ± 4.9	–	14.7 ± 3.5	IC	21	54.5 ± 9.4	–	36.6 ± 4.9	–	14.7 ± 3.5
Smith et al. (63)	UK	RCT	Double	6 weeks	IC	26	61 ± 8	88.5	31 ± 4	36 ± 23	10 ± 5	CPAP	26	61 ± 8	88.5	31 ± 4	36 ± 23	10 ± 5
Patruno et al. (64)	Italy	RCT	–	3 months	APAP	15	46.7 ± 11.6	80	36.4 ± 7.1	47.3 ± 14.7	15.8 ± 3.5	CPAP	16	47 ± 10.7	81.2	36.4 ± 7.1	47.3 ± 14.7	15.8 ± 3.5
Martinez-Garcia et al. (65)	Spain	–	–	3 months	CPAP	10	68.1 ± 7.8	52.2	35.1 ± 5.2	40 ± 19.7	7.4 ± 5.1	IC	23	72.2 ± 3.2	50	35.1 ± 5.2	40 ± 19.7	7.4 ± 5.1
Lam et al. (66)	China	RCT	–	10 weeks	CPAP	34	45 ± 1	79	27.6 ± 3.5	23.8 ± 11.1	12 ± 5.8	OA	34	45 ± 2	76	27.6 ± 3.5	23.8 ± 11.1	12 ± 5.8
Haensel et al. (67)	Netherlands	RCT	Double	2 weeks	CPAP	25	48.2 ± 10.2	80	33.1 ± 8.2	63.6 ± 29.1	–	IC	25	49.0 ± 10.6	80	33.1 ± 8.2	63.6 ± 29.1	–
Fietze et al. (68)	Germany	RCT	–	6 weeks	APAP	10	56.9 ± 9.3	100	32.6 ± 6.6	43.3 ± 30.2	–	CPAP	11	51.8 ± 13.5	90.9	32.6 ± 6.6	43.3 ± 30.2	–
Drager et al. (69)	Brazil	RCT	Double	4 months	IC	12	47 ± 6	–	29.7 ± 2.9	62 ± 22	13 ± 5	CPAP	12	44 ± 7	–	29.7 ± 2.9	62 ± 22	13 ± 5
Coughlin et al. (70)	UK	RCT	Single	6 weeks	CPAP	34	49.0 ± 8.3	–	36.1 ± 7.6	–	13.8 ± 4.9	IC	34	49.0 ± 8.3	–	36.1 ± 7.6	–	13.8 ± 4.9
Robinson et al. (71)	UK	RCT	Open-label	1 months	IC	16	54 ± 8	86.1	33.2 ± 5.3	–	5.3 ± 1	CPAP	16	54 ± 8	86.1	33.2 ± 5.3	–	5.3 ± 1
Hui et al. (72)	China	RCT	Single	3 months	CPAP	28	50.3 ± 1.6	78.6	27.5 ± 3.2	32.9 ± 16.9	10.7 ± 5.3	IC	28	51.2 ± 1.8	75	27.5 ± 3.2	32.9 ± 16.9	10.7 ± 5.3
Campos-Rodriguez et al. (73)	Spain	RCT	Double	1 months	CPAP	34	55.3 ± 9.6	55.8	35.7 ± 5.6	58.3 ± 24.6	15 ± 3.9	IC	34	58.0 ± 7.0	64.7	35.7 ± 5.6	58.3 ± 24.6	15 ± 3.9
Usui et al. (74)	Canada	RCT	–	1 months	OA	9	52.2 ± 4.1	77.8	31.3 ± 4.8	–	–	CPAP	8	55.0 ± 2.0	100	31.3 ± 4.8	–	–
Marshall et al. (75)	New Zealand	RCT	Double	3 weeks	IC	29	45 ± 9.8	75.9	32.3 ± 3.5	–	12.5 ± 4.3	CPAP	29	45 ± 9.8	75.9	32.3 ± 3.5	–	12.5 ± 4.3
Blanco et al. (76)	Spain	RCT	–	2 weeks	OA	8	55.6 ± 11.8	86.7	27.9 ± 4.3	33.8 ± 14.7	–	IC	7	55.6 ± 11.8	86.7	27.9 ± 4.3	33.8 ± 14.7	–
Arias et al. (77)	Spain	RCT	Double	3 months	IC	25	52 ± 13	100	30.5 ± 4	44 ± 27.5	–	CPAP	25	52 ± 13	100	30.5 ± 4	44 ± 27.5	–
Masa et al. (78)	Spain	RCT	Single	3 months	CPAP	126	51.0 ± 9.1	86.9	33.6 ± 8.4	61.8 ± 22	15.9 ± 3.5	APAP	119	52.2 ± 10.4	89.6	33.6 ± 8.4	61.8 ± 22	15.9 ± 3.5
Mansfield et al. (79)	Australia	RCT	Single	3 months	IC	21	57.5 ± 1.6	88.9	33.3 ± 5.5	26.6 ± 20.6	8.8 ± 4.1	CPAP	19	57.2 ± 1.7	100	33.3 ± 5.5	26.6 ± 20.6	8.8 ± 4.1
Lloberes et al. (80)	Spain	–	–	3 months	APAP	27	53.9 ± 7.7	81.5	32 ± 5.8	55.2 ± 24.2	13.3 ± 5.1	CPAP	30	58.6 ± 8.7	76.7	32 ± 5.8	55.2 ± 24.2	13.3 ± 5.1
Ip et al. (81)	China	RCT	Single	3 months	CPAP	14	44.4 ± 6.9	100	29.6 ± 5.7	47.7 ± 15.3	–	IC	13	40.9 ± 11.1	100	29.6 ± 5.7	47.7 ± 15.3	–
Hussain et al. (82)	Pakistan	RCT	Single	1 week	APAP	10	44.9 ± 9.7	90	35.9 ± 12.9	47.2 ± 35.6	11.1 ± 6.4	CPAP	10	44.9 ± 9.7	90	35.9 ± 12.9	47.2 ± 35.6	11.1 ± 6.4
Gotsopoulos et al. (83)	Australia	RCT	Double	1 month	IC	33	48 ± 11	79.1	29.2 ± 27.6	27 ± 86.2	–	OA	31	48 ± 11	79.1	29.2 ± 27.6	27 ± 86.2	–
Barnes et al. (84)	Australia	RCT	–	3 months	CPAP	97	47.0 ± 0.9	78.38	31 ± 5.9	21.3 ± 12.8	10.7 ± 3.9	OA	99	47.0 ± 0.9	78.38	31 ± 5.91	21.3 ± 12.8	10.7 ± 3.9
Woodson et al. (85)	USA	RCT	Double	6 months	IC	30	46.0 ± 8.1	70	28.5 ± 4.2	15.4 ± 7.8	11.6 ± 3.5	CPAP	28	51.7 ± 8.6	75	28.5 ± 4.2	15.4 ± 7.8	11.6 ± 3.5
Kaneko et al. (86)	Japan	RCT	Single	1 month	IC	12	55.2 ± 3.6	83.3	32.3 ± 8.7	45.2 ± 18.3	5.7 ± 3.1	CPAP	12	55.9 ± 2.5	91.7	32.3 ± 8.7	45.2 ± 18.3	5.7 ± 3.1
Becker et al. (87)	Australia	RCT	Single	3 months	CPAP	16	54.4 ± 8.9	93.7	33.3 ± 5.1	62.5 ± 17.8	14.4 ± 2.5	IC	16	52.3 ± 8.4	87.5	33.3 ± 5.1	62.5 ± 17.8	14.4 ± 2.5
Tan et al. (88)	UK	RCT	–	2 months	CPAP	10	50.9 ± 10.1	83.3	31.9 ± 6.8	22.2 ± 9.6	13.4 ± 4.6	OA	14	50.9 ± 10.1	83.3	31.9 ± 6.8	22.2 ± 9.6	13.4 ± 4.6
Randerath et al. (89)	Germany	RCT	–	6 weeks	CPAP	20	56.5 ± 10.2	80	31.2 ± 6.4	17.5 ± 7.7	–	OA	20	56.5 ± 10.2	80	31.2 ± 6.4	17.5 ± 7.7	–
Pepperell et al. (90)	UK	RCT	Double	4 weeks	IC	59	51.0 ± 9.8	–	35.3 ± 6	–	16 ± 3.1	CPAP	59	50.1 ± 10.4	–	35.3 ± 6	–	16 ± 3.1
Gotsopoulos et al. (91)	Australia	RCT	Double	3 months	OA	73	48 ± 11	80.8	29 ± 4.5	–	–	IC	73	48 ± 11	80.8	29 ± 40.1	–	–
Monasterio et al. (92)	Spain	RCT	Single	6 months	IC	59	54 ± 9	91	29.5 ± 3.3	21 ± 6	13.2 ± 4.3	CPAP	66	53 ± 9	81	29.5 ± 3.3	21 ± 6	13.2 ± 4.3
Bardwell et al. (93)	USA	RCT	–	1 week	IC	16	48 ± 2.2	–	29.6 ± 5.2	–	–	CPAP	20	47 ± 1.9	–	29.6 ± 5.2	–	–
Barbé et al. (94)	Spain	RCT	Single	6 weeks	CPAP	29	54 ± 2	89.6	29 ± 5.4	54 ± 16.1	7 ± 2.1	IC	25	52 ± 2	96	29 ± 5.4	54 ± 16.1	7 ± 2.1
Ballester et al. (95)	Spain	RCT	–	3 months	IC	37	54 ± 1.5	86.5	34 ± 7.3	58 ± 18.2	11.4 ± 6.1	CPAP	68	53 ± 1.3	88.2	34 ± 7.3	58 ± 18.2	11.4 ± 6.1
Redline et al. (96)	USA	RCT	–	2 months	IC	46	49.2 ± 10.5	–	32 ± 8.5	–	10.6 ± 5.6	CPAP	51	48.1 ± 9.2	–	32 ± 8.5	–	10.6 ± 5.6
Ferguson et al. (97)	Canada	RCT	–	3 months	OA	24	44 ± 10.6	–	32 ± 8.2	25.3 ± 15.0	–	CPAP	24	44 ± 10.6	–	32 ± 8.2	25.3 ± 15	–
Meurice et al. (98)	Canada	RCT	Double	3 weeks	APAP	8	54 ± 11	100	34.2 ± 5.7	40.5 ± 17.7	15.2 ± 4.2	CPAP	8	54 ± 11	100	34.2 ± 5.7	40.5 ± 17.7	15.2 ± 4.2
Ferguson et al. (99)	Canada	RCT	–	4 months	OA	25	46.2 ± 10.9	88.9	30.4 ± 4.8	19.7 ± 13.8	–	CPAP	21	46.2 ± 10.9	88.9	30.4 ± 4.8	19.7 ± 13.8	–

*Treatment: CPAP, continuous positive airway pressure; APAP, auto-adjusting positive airway pressure; IC, inactive control; OA, oral appliance*.

*Baseline: BMI, body mass index; AHI, Apnea–Hypopnea Index; ESS, Epworth Sleepiness Scale*.

### Meta-analysis Result

We summarized pair-wise comparisons from MA and details of available data were shown in Table 2. When compared with IC, CPAP showed better efficacy regarding all outcomes with WMD = 23.82, 95% CI: (18.49, 29.15) for AHI, WMD = 2.07, 95% CI: (1.51, 2.62) for ESS, WMD = 10.76, 95% CI: (7.84, 13.69) for AI, and WMD = −9.35, 95% CI: (−11.41, −7.29) for SaO_2_. In addition, CPAP could significantly reduce blood pressure than IC [WMD = 1.89, 95% CI: (0.86, 2.92) for 24 h SBP; WMD = 1.70, CI: (1.13, 2.27) for 24 h DBP; WMD = 3.09, 95% CI: (2.18, 4.01) for dSBP; WMD = 1.98, 95% CI: (1.25, 2.70) for dDBP; and WMD = 4.22, 95% CI: (2.14, 6.30) for nSBP; WMD = 1.97, 95% CI: (0.81, 3.13) for nDBP]. No statistical difference occurred in the comparison of CPAP versus APAP. However, CPAP presented greater reduction than OA in AHI [WMD = −8.77, 95% CI: (−16.05, −1.50)] and AI [WMD = −2.59, 95% CI: (−4.91, −0.27)], as well as blood pressure [WMD = −9.57, 95% CI: (−11.34, −7.81) for 24 h SBP; WMD = −7.11, 95% CI: (−8.06, −6.15) for 24 h DBP; WMD = −7.87, 95% CI: (−13.28, −2.46) for dSBP], and it led to an increase in SaO_2_ (WMD = 4.91, 95% CI: 2.85–6.97) versus OA. We found significant improvement with treatment of OA compared with IC, in the outcomes of AHI [WMD = 9.40, 95% CI: (5.57, 13.23)], ESS [WMD = 1.15, 95% CI: (0.43, 1.87)], AI [WMD = 9.16, 95% CI: (2.70, 15.61)], SaO_2_ [WMD = −2.57, 95% CI: (−3.53, −1.61)], 24 h SBP [WMD = 1.64, 95% CI: (0.02, 3.26)], and dDBP [WMD = 2.15, 95% CI: (0.02, 4.28)]. Generally, according to the MA results, CPAP and OA were proved to be more efficacious than IC, while there were no obvious difference in the effectiveness of CPAP and APAP. CPAP showed higher ability of reducing AHI, AI, and blood pressure than OA.

**Table 2:** Direct pair-wise comparison results of obstructive sleep apnea.

Outcomes	IC versus CPAP		CPAP versus APAP		CPAP versus OA		IC versus OA	
	WMD (95% CI)	*I*^2^ (%)	WMD (95% CI)	*I*^2^ (%)	WMD (95% CI)	*I*^2^ (%)	WMD (95% CI)	*I*^2^ (%)
AHI	23.82 (18.49, 29.15)	93.2	0.10 (−3.15, 3.34)	0.0	−8.77 (−16.05, −1.50)	96.2	9.40 (5.57, 13.23)	32.7
ESS	2.07 (1.51, 2.62)	88.0	0.08 (−0.68, 0.85)	12.7	−0.31 (−1.02, 0.39)	0.0	1.15 (0.43, 1.87)	0.0
AI	10.76 (7.84, 13.69)	14.9	0.32 (−3.05, 3.69)	0.0	−2.59 (−4.91, −0.27)	0.0	9.16 (2.70, 15.61)	78.9
SaO_2_	−9.35 (−11.41, −7.29)	56.8	2.71 (−0.88, 6.29)	0.0	4.91 (2.85, 6.97)	60.0	−2.57 (−3.53, −1.61)	0.0
24 h SBP	1.89 (0.86, 2.92)	23.3	–	–	−9.57 (−11.34, −7.81)	0.0	1.64 (0.02, 3.26)	0.0
24 h DBP	1.70 (1.13, 2.27)	0.0	–	–	−7.11 (−8.06, −6.15)	0.0	1.18 (−0.02, 2.38)	0.0
dSBP	3.09 (2.18, 4.01)	6.8	−1.70 (−5.11, 1.71)	–	−7.87 (−13.28, −2.46)	56.5	2.81 (−0.13, 5.74)	0.0
dDBP	1.98 (1.25, 2.70)	14.4	−1.30 (−3.47, 0.87)	–	−2.40 (−8.01, 3.26)	–	2.15 (0.02, 4.28)	0.0
nSBP	4.22 (2.14, 6.30)	68.0	−1.10 (−4.74, 2.54)	–	−3.90 (−12.88, 5.08)	–	1.06 (−2.09, 4.21)	0.0
nDBP	1.97 (0.81, 3.13)	45.9	−1.30 (−3.42, 0.82)	–	−2.10 (−7.80, 3.60)	–	0.95 (−1.23, 3.14)	0.0

*Treatment: CPAP, continuous positive airway pressure; APAP, auto-adjusting positive airway pressure; IC, inactive control; OA, oral appliance*.

*Outcomes: AHI, Apnea–Hypopnea Index; ESS, Epworth Sleepiness Scale; SaO_2_, arterial oxygen saturation; AI, arousal index; SBP, systolic blood pressure; DBP, diastolic blood pressure; d, daytime; n, nighttime; WMD, weighted mean difference; CI, confidence interval*.

### Network Meta-analysis

**Figure 2:**
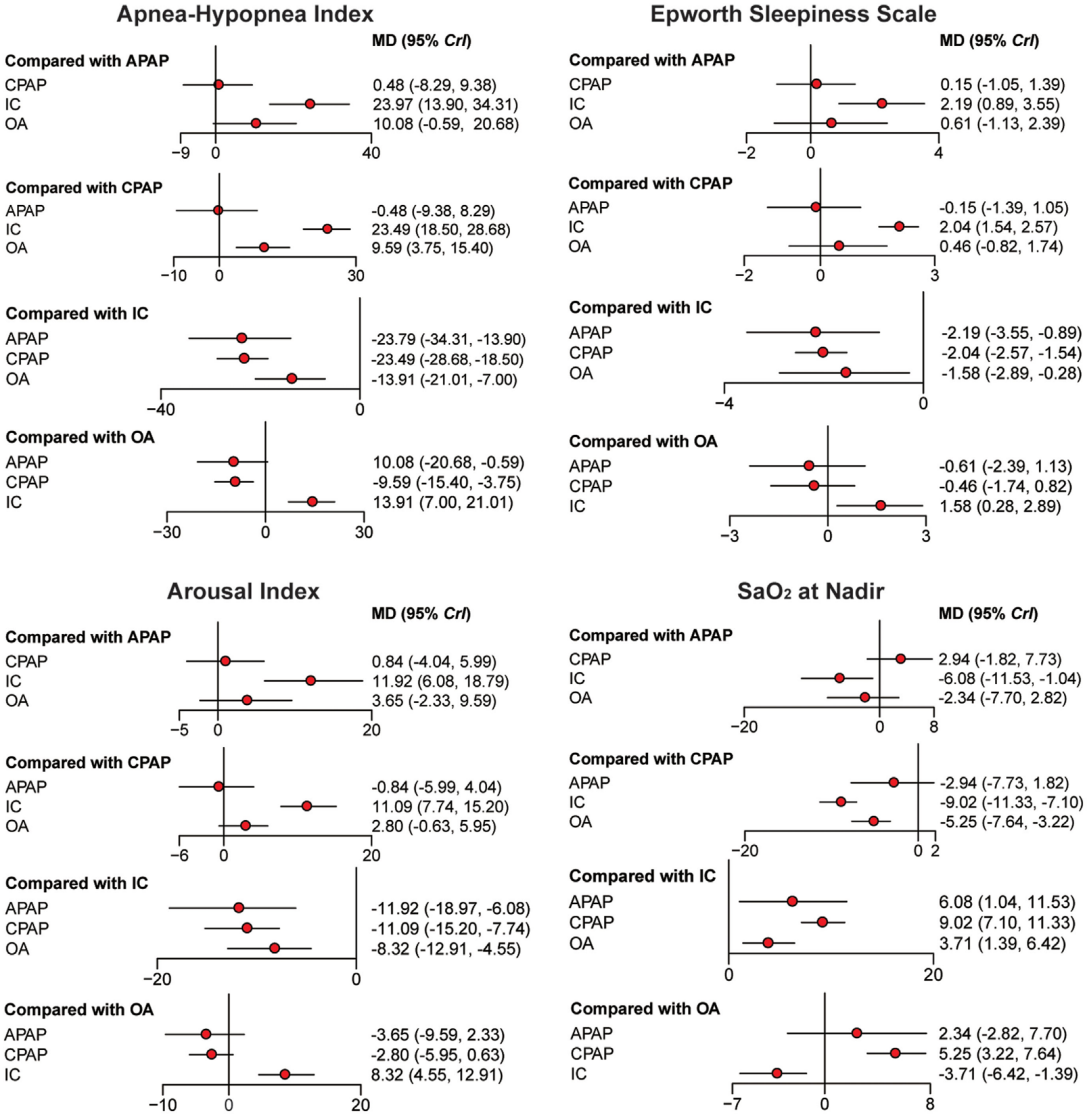
Forest plots regarding Apnea–Hypopnea Index, Epworth Sleepiness Scale, arousal index, and SaO_2_. Mean difference (MD) with 95% credible interval (CrIs) indicate the relative efficacy. Abbreviations: CPAP, continuous positive airway pressure; APAP, auto-adjusting positive airway pressure; IC, inactive control; OA, oral appliance.

**Figure 3:**
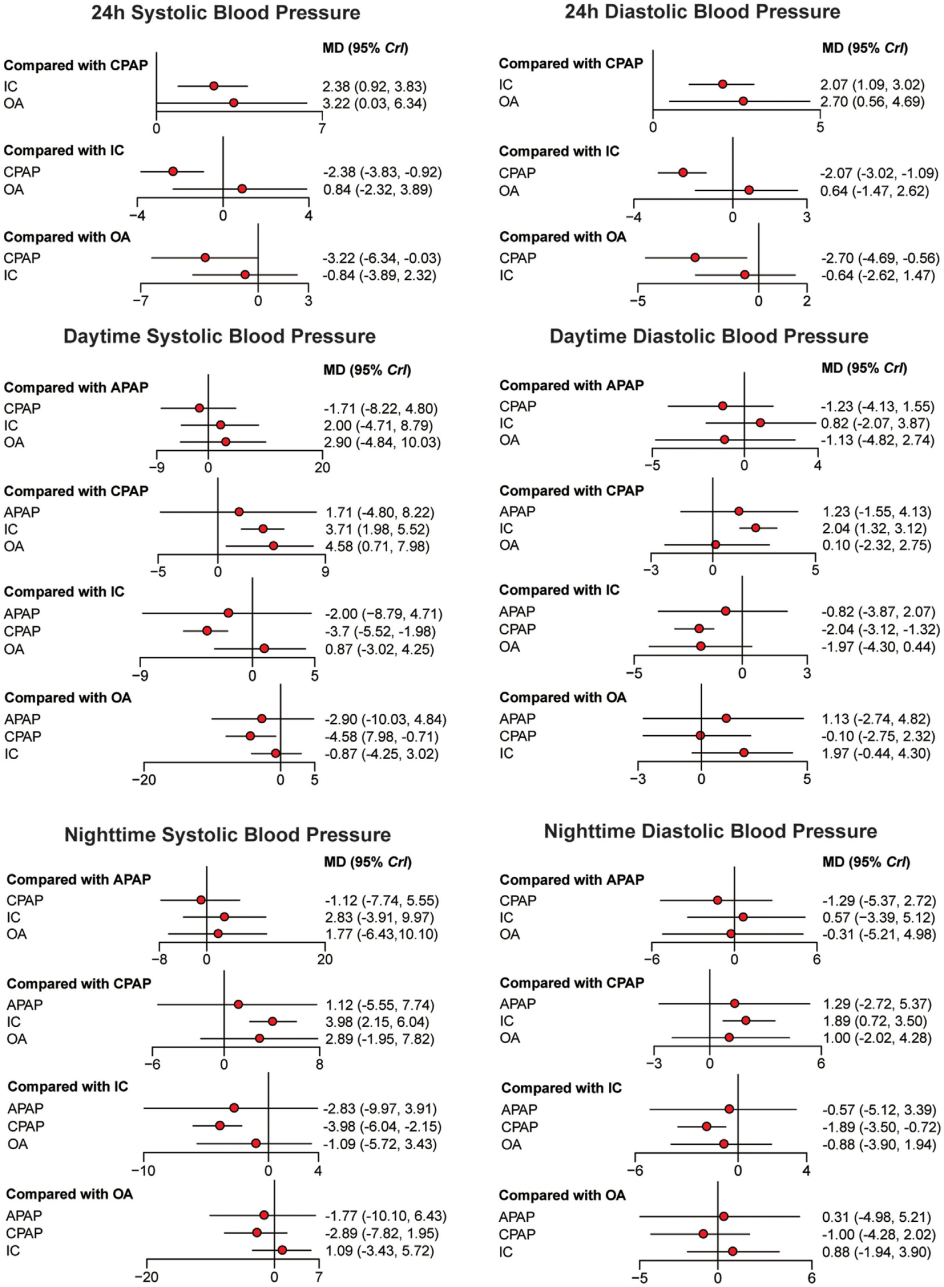
Forest plots regarding 24 h systolic blood pressure (SBP), 24 h diastolic blood pressure, daytime SBP, daytime SBP, nighttime SBP, and nighttime SBP. Mean difference (MD) with 95% credible interval (CrIs) indicate the relative efficacy. Abbreviations: CPAP, continuous positive airway pressure; APAP, auto-adjusting positive airway pressure; IC, inactive control; OA, oral appliance.

Bayesian models allowed for more refined estimates of efficacy when participants were treated with APAP, CPAP, OA, and IC. Available data was recorded in Table 3 and displayed graphically in the forest plots in Figure 2 for sleep characteristics and Figure 3 for blood pressure. For the primary outcomes of AHI, APAP, CPAP, and OA all showed statistically reduction versus IC [mean difference (MD) = −23.97, 95% CrI: (13.90, 34.31) for APAP; MD = −23.49, 95% CrI: (−28.68, −18.50) for CPAP; MD = −13.91, 95% CrI: (−21.01, −7.00) for OA]. Significant decrease of AHI occurred in comparison of CPAP versus OA [MD = −9.59, 95% CrI: (−15.40, −3.75) for CPAP]. Similarly, statistical significance was observed in ESS for APAP, CPAP, and OA compared with IC, with MD = 2.19, 95% CrI: (0.89, 3.55) for APAP; MD = −2.04, 95%CrI: (1.54, 2.57) for CPAP; and MD = −1.58 95% CrI: (−2.89, −0.28) for OA. Similar observation was obtained in AI, with treatments of the three active interventions [MD = 11.92, 95% CrI: (6.09, 18.97) for APAP versus IC; MD = 11.09, 95%CrI: (7.74, 15.20) for CPAP versus IC; MD = −8.32, 95% CrI: (−12.91, −4.55) for IC versus OA]. Increase in SaO_2_ was exhibited in compares with IC [MD = −6.08, 95% CrI: (−11.53, −1.04) for APAP versus IC; MD = −9.02, 95% CrI: (−11.33, −7.10) for CPAP versus IC; MD = 3.71, 95% CrI: (1.39, 6.42) for IC versus OA]. In addition, we found great difference between OA and CPAP concerning SaO_2_ [MD = −5.25, 95% CrI: (−7.64, −3.22)]. For the secondary outcomes of blood pressure, more negative results were obtained. Data in 24 h SBP and 24 h DBP was merely available among CPAP, OA, and IC. Similar statistically significant results were presented in 24 h SBP [MD = 2.38, 95% CrI: (0.92, 3.83) for IC; MD = 3.22, 95% CrI: (0.03, 6.34) for OA] and 24 h DBP [MD = 2.07, 95% CrI: (1.09, 3.02) for IC; MD = 2.70, 95% CrI: (0.56, 4.69) for OA] when comparing with CPAP. We got complete information of all the interventions in terms of SBP and DBP during daytime and nighttime. A high similarity among the four plots was founded. CPAP contributed to significant reduction in daytime SBP [MD = 3.70, 95% CrI: (1.98, 5.52) for IC versus CPAP], daytime DBP [MD = 2.04, 95% CrI: (1.32, 3.12) for IC versus CPAP], nighttime SBP [MD = 3.98, 95% CrI: (2.15, 6.04) for IC versus CPAP] and nighttime DBP [MD = −1.89, 95% CrI: (−3.50 to −0.72) versus IC]. A comparison of OA versus CPAP in daytime SBP was also noted for indicating significant difference [MD = 4.58, 95% CrI: (0.71, 7.98)]. In all, based on the network results of primary outcomes, namely, AHI and ESS, significant improvement of APAP, CPAP, and OA were observed compared with IC, outcomes were at least in favor of CPAP when compared with OA, and APAP and CPAP could be classified as identical.

**Table 3:** Network meta-analysis results of obstructive sleep apnea.

		APAP	CPAP	IC	OA	
ESS	APAP	1	0.48 (−8.29, 9.38)	**23.97 (13.90, 34.31)**	10.08 (−0.59, 20.68)	AHI
	CPAP	0.15 (−1.05, 1.39)	**1**	**23.49 (18.50, 28.68)**	**9.59 (3.75, 15.40)**	
	IC	**2.19 (0.89, 3.55)**	**2.04 (1.54, 2.57)**	**1**	**−13.91 (−21.01, −7.00)**	
	OA	0.61 (−1.13, 2.39)	0.46 (−0.82, 1.74)	**−1.58 (−2.89, −0.28)**	1	

SaO_2_	APAP	1	0.84 (−4.04, 5.99)	**11.92 (6.08, 18.79)**	3.65 (−2.33, 9.59)	AI
	CPAP	2.94 (−1.82, 7.73)	1	**11.09 (7.74, 15.2)**	2.80 (−0.63, 5.95)	
	IC	**−6.08 (−11.53, −1.04)**	**−9.02 (−11.33, −7.10)**	1	**−8.32 (−12.91, −4.55)**	
	OA	−2.34 (−7.70, 2.82)	**−5.25 (−7.64, −3.22)**	**3.71 (1.39, 6.42)**	1	

24 h DBP	CPAP	–	1	**2.38 (0.92, 3.83)**	**3.22 (0.03, 6.34)**	24 h SBP
	IC	–	**2.07 (1.09, 3.02)**	1	0.84 (−2.32, 3.89)	
	OA	–	**2.7 (0.56, 4.69)**	0.64 (−1.47, 2.62)	1	

Daytime DBP	APAP	1	**−1.71 (−8.22, 4.80)**	2.00 (−4.71, 8.79)	2.90 (−4.84, 10.03)	Daytime SBP
	CPAP	−1.23 (−4.13, 1.55)	1	**3.71 (1.98, 5.52)**	**4.58 (0.71, 7.98)**	
	IC	0.82 (−2.07, 3.87)	**2.04 (1.32, 3.12)**	1	0.87 (−3.02, 4.25)	
	OA	−1.13 (−4.82, 2.74)	0.1 (−2.32, 2.75)	−1.97 (−4.30, 0.44)	1	

Nighttime DBP	APAP	1	−1.12 (−7.74, 5.55)	2.83 (−3.91, 9.97)	1.77 (−6.43, 10.10)	Nighttime SBP
	CPAP	−1.29 (−5.37, 2.72)	1	**3.98 (2.15, 6.04)**	2.89 (−1.95, 7.82)	
	IC	0.57 (−3.39, 5.12)	**1.89 (0.72, 3.50)**	1	−1.09 (−5.72, 3.43)	
	OA	−0.31 (−5.21, 4.98)	1.00 (−2.02, 4.28)	−0.88 (−3.90, 1.94)	1	

*Treatment: CPAP, continuous positive airway pressure; APAP, auto-adjusting positive airway pressure; IC, inactive control; OA, oral appliance*.

*Outcomes: AHI, Apnea–Hypopnea Index; ESS, Epworth Sleepiness Scale; SaO_2_, arterial oxygen saturation; AI, arousal index; SBP, systolic blood pressure; DBP, diastolic blood pressure*.

*Bold font indicates statistically significant difference*.

### Ranking Scheme Based on SUCRA

**Figure 4:**
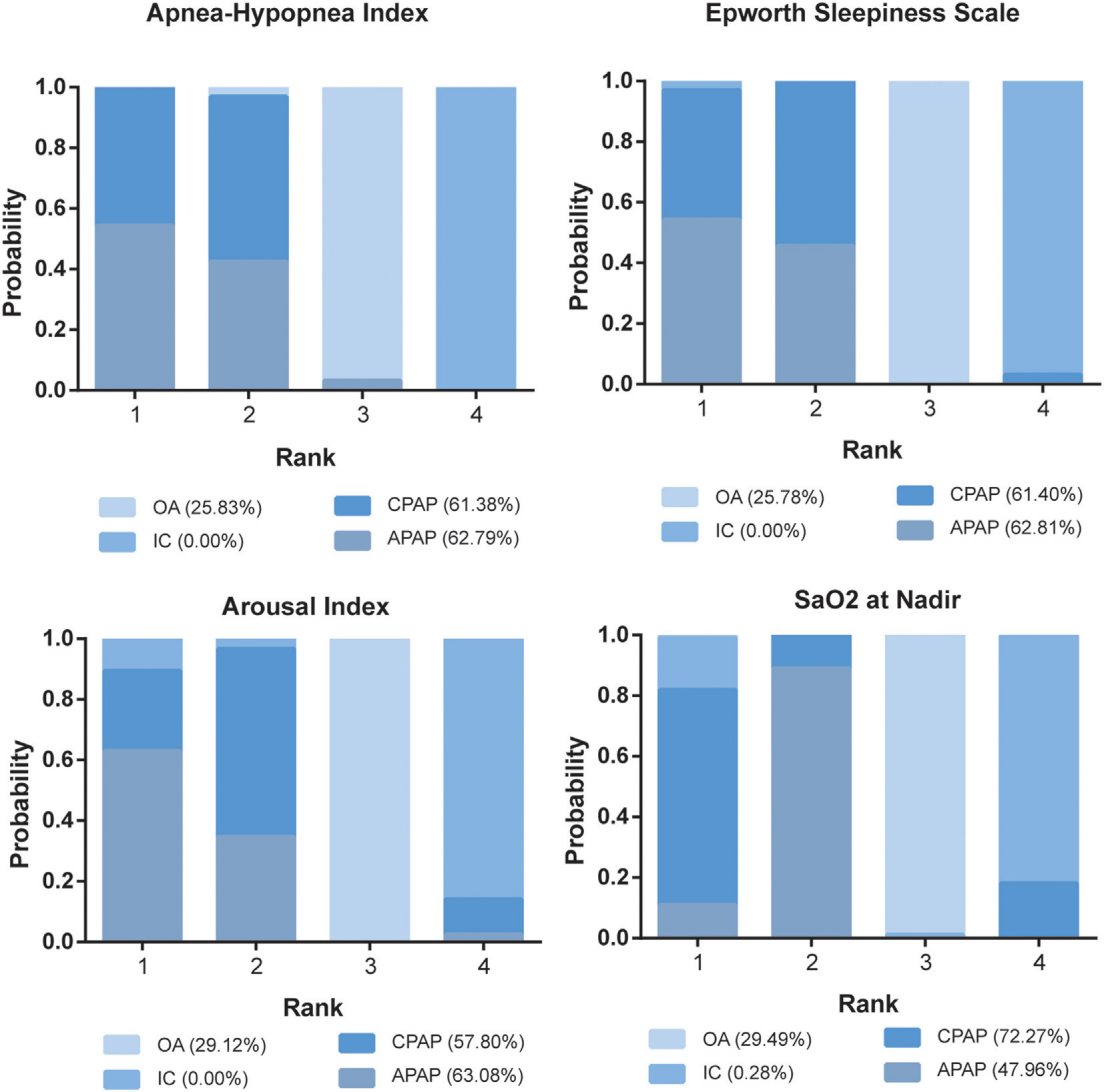
Stacked bar charts showing the rankings of four therapies for Apnea–Hypopnea Index, Epworth Sleepiness Scale, arousal index, and SaO_2_ at Nadir. The percentage number included in each pair of brackets indicates the cumulative ranking probability of the corresponding therapy. Abbreviations: CPAP, continuous positive airway pressure; APAP, auto-adjusting positive airway pressure; IC, inactive control; OA, oral appliance.

**Figure 5:**
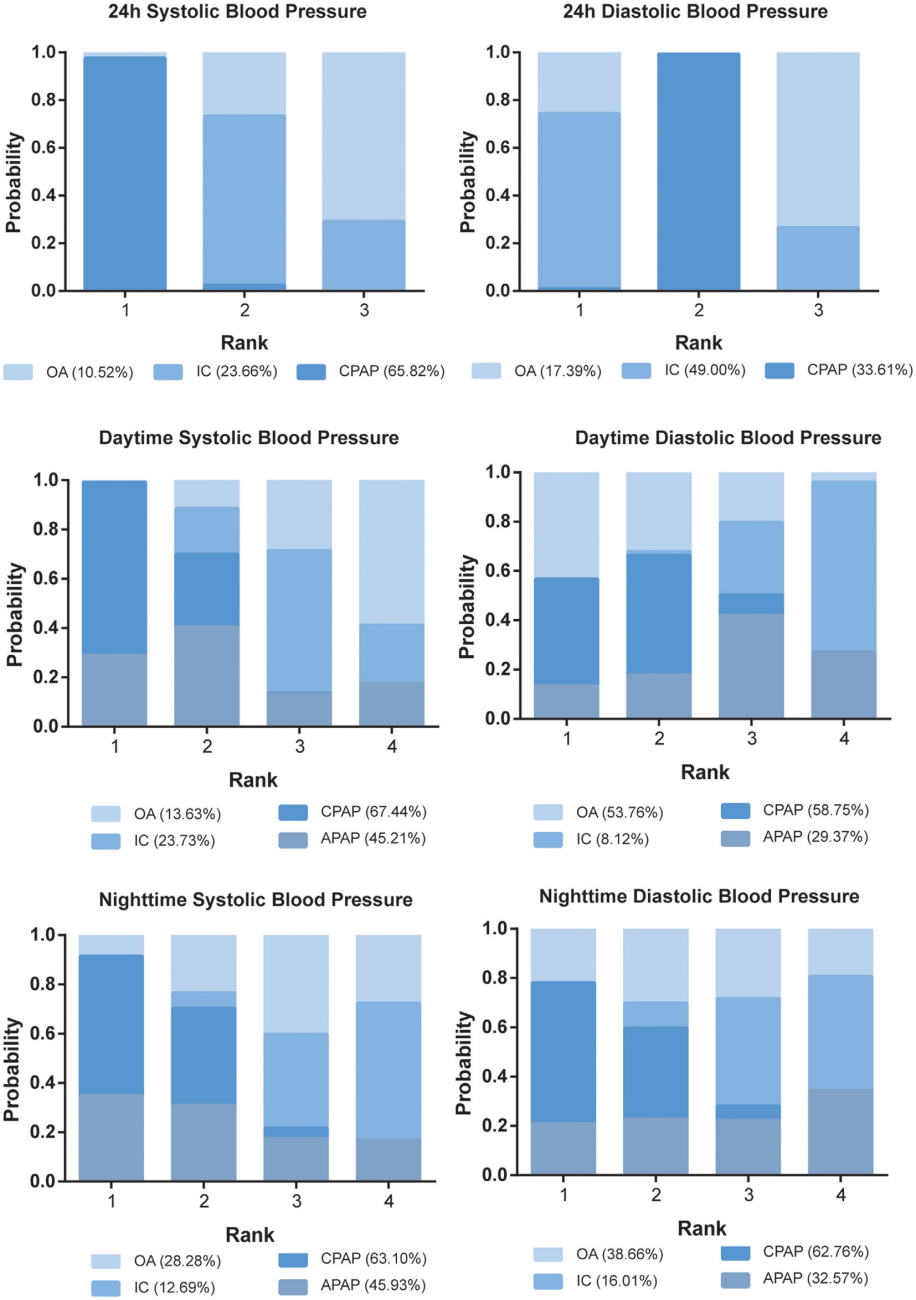
Stacked bar charts showing the rankings of four therapies for 24 h systolic blood pressure (SBP), 24 h diastolic blood pressure, daytime SBP, daytime SBP, nighttime SBP, and nighttime SBP. The percentage number included in each pair of brackets indicates the cumulative ranking probability of the corresponding therapy. Abbreviations: CPAP, continuous positive airway pressure; APAP, auto-adjusting positive airway pressure; IC, inactive control; OA, oral appliance.

The ranking probability of each treatment in terms of 10 outcomes was illustrated in Figures 4 and 5. CPAP and APAP were ranked top two in improving sleep characteristics, with similar ranking score in AHI (61.38% for CPAP and 62.79% for APAP) and ESS (61.40% for CPAP and 62.81% for APAP), best for APAP in AI (63.08%) and best for CPAP in SaO_2_ (72.27%). CPAP kept ranking as number one in reducing blood pressure and APAP became moderate. OA was regarded as a mild intervention, and IC was the last choice under all the circumstances. CPAP was recommended based on the ranking results.

### Risk of Bias and Consistency

Jadad scale of included studies was presented in Table S1 in Supplementary Material, which indicated medium–high quality and low risk of publication bias for all included studies. And the symmetry of the “comparison adjusted” funnel plots in Figures S1 and S2 in Supplementary Material had suggested that there was no remarkable publication bias. According to the node-splitting plots in Figure S3 in Supplementary Material, all the *P*-values were higher than 0.05, suggesting no significant inconsistency in terms of sleep characteristics. However, great inconsistency was obtained in the comparison of CPAP versus IC with respect to blood pressure, which indicated that indirect evidence had higher extent in reducing 24 h blood pressure, and the comparison of OA versus IC, in which indirect evidence provided adverse results (Figure S4 in Supplementary Material). Furthermore, the *P*-values showed that there was no consistency in the comparisons among CPAP, OA, and IC. The result of assessment for consistency was displayed in Figures S5 and S6 in Supplementary Material.

## Discussion

In this study, NMA regarding AHI, ESS, AI, SaO_2_, and blood pressures was performed to evaluate the efficacy of CPAP, APAP, and OA in OSA patients. OSA is a detrimental disease since it results in not only sleepiness and snoring but also significant health problems such as atrial fibrillation (100).

As a first line therapy for OSA, CPAP was first recommended by the American College of Physicians (100), and Wright and White proposed in 2000 that the effect of CPAP on sleepiness is clinically significant since CPAP therapy is able to improve the life quality of OSA patients (101). Xu et al. confirmed that CPAP could also decrease the total cholesterol level, especially for younger and more obese patients who use CPAP in the long term, while the issue of lipid metabolism was not clinically significant (102).

Our NMA confirmed previous findings that CPAP is effective in improving AHI, ESS, AI, and SaO_2_. Ha et al. revealed that CPAP is superior to positional therapy in reducing the severity of sleep apnea (103). Previous studies have revealed that CPAP are capable of preventing upper airway collapse and arousals, as well as reducing oxidative stresses. Besides that, CPAP therapy can alleviate the corresponding symptoms of OSA such as excessive daytime sleepiness and snoring (104, 105). As Liu et al. concluded, CPAP was associated with significant reductions in SBP, DBP, and nocturnal DBP in patients with OSA and hypertension. Our research further revealed the positive effects of CPAP on nighttime DBP and daytime blood pressures (106). CPAP therapy significantly reduces BP in patients with OSA but the effect size may not be clinically significant. APAP presented a similar efficacy to CPAP on improving sleeping quality and reducing daytime sleepiness, since the comparisons between CPAP and APAP on AHI and ESS manifest neither statistical difference nor distinctive preferences. The above result is supported by Gao et al. who concluded that the effect of APAP on AHI improvements was identical to standard manual titration (10). Gao et al. also pointed out that the acceptance and compliance of automatic titrated method had the same performance as manual titration. Since APAP has the potential advantage in saving time and cost, automatic titration was also recommended as an alternative therapy in clinical practice (10). Despite we concluded that CPAP and APAP are identical in clinical outcomes, Xu et al. claimed that the use of APAP is slightly favored compared with fixed pressure CPAP in some aspects, such as compliance and patient preference, though the clinical relevance still requires further study (107). Despite the efficacy of APAP in sleeping quality, blood pressure outcomes are in favor of CPAP based on the SUCRA result, this preference might be the result of the relatively lower average pressure that APAP applies to OSA patients.

Similar to CPAP, OA exerts its function by relieving upper airway collapse during sleep through the modification of the position of mandible, tongue, and pharyngeal structures (58). Though inferior to CPAP, OA was also confirmed to be effective in improving symptoms and life qualities for patients with OSA. Okuno et al. supported our notion that compared with untreated patients, significant reduction in AHI and AI was found after OA therapy was applied (14). Due to its efficacy and cost saving features, OA has become increasingly popular and performed its clinical application as alternative therapy for CPAP (58). Yet, it should also be noted that the efficacy of OA is directly related to its type (18). Umemoto et al. found that fixed OAs are superior in treating OSA than twin-block appliances because of their ability to prevent mouth opening and reduce incisal overjet (108).

A high inconsistency between direct and indirect evidence was observed regarding the outcome of 24 h SBP, 24 h DBP, and daytime SBP, which concurs with Liu et al. that the beneficial effects of CPAP are inconsistent (106). Despite our conclusion that CPAP could significantly reduce all blood pressure outcomes, there existed studies that claimed non-significant decrease in BP was found (109, 110). This inconsistency might come from the discordance of OSA patient baseline characteristics and CPAP uses. Since studies reached agreement that the efficacy of CPAP increases with the severity of OSA, frequent apneic episodes may benefit the most from CPAP (111). Based on these conclusions, it is highly possible that results from different studies may vary due to the discordance in the baseline characteristics of different patients.

Our NMA originally combined conclusions on sleeping behavior and blood pressure; thus, a more overall efficacy of different therapy could be drawn. However, there also existed some limitations. Though an amount of 84 studies were included in our NMA, the sample size is still limited and resulted in the inconsistency discussed above. The included studies also presented deficient comparison between different therapy, such as APAP and OA. Moreover, the baseline of studies should be more unified to ensure the credibility and accuracy of our conclusions. Thus, larger comparison with better designed clinical trials is still required for a more comprehensive conclusion.

In all, CPAP, APAP, and OA are proved to be effective, which is supported by previous evidences. Based on primary outcomes, namely, AHI and ESS, significant improvement was observed compared with IC, outcomes are at least in favor of CPAP when compared with OA, and APAP and CPAP are classified as identical. Apart from ESS that represents reduction in daytime sleepiness, CPAP also presented significant improvements with respect to secondary outcomes like blood pressure. APAP tended to have slightly better performance than CPAP in AHI and ESS but are less promising in blood pressures on the basis of SUCRA. Our NMA identified CPAP as most efficacious treatment for OSA patients after synthetically evaluation on ESS, AHI, AI, SaO_2_, and blood pressures. Though inferior to CPAP and exerted no distinctive benefits on blood pressure, OA still manifested significant improvements in AHI and ESS compared with IC, indicating its feasibility as an alternative therapy for OSA patients. Larger clinical trials on the efficacy of CPAP on blood pressure for patients with OSA are needed for further investigation on the inconsistency observed.

## Author Contributions

TL performed the research and designed the research study; WL analyzed and interpreted the data and wrote the paper; HZ drafted the manuscript; ZW made critical revision of the manuscript. All authors approved the final manuscript.

## Conflict of Interest Statement

The authors declare that the research was conducted in the absence of any commercial or financial relationships that could be construed as a potential conflict of interest.
