# Supplementary material for: Verifying the Relative Efficacy between Continuous Positive Airway Pressure Therapy and Its Alternatives for Obstructive Sleep Apnea: A Network Meta-analysis
Source: Front Neurol. 2017 Jun 28;8:289. doi: 10.3389/fneur.2017.00289 (PMC5487413; doi:10.3389/fneur.2017.00289)
Supplement: Supplementary file 1 [file Table_1.DOCX]

**Table S1. Quality score of included studies using Jadad Scale**

| **Author, Year** | **Randomization** | **Blinding** | **Withdrawal** |
| --- | --- | --- | --- |
| Salord, 2016 | 2 | 2 | 1 |
| Pépin, 2016 | 2 | 2 | 1 |
| Paz, 2016 | 2 | 2 | 0 |
| Pamidi, 2015 | 2 | 0 | 0 |
| Muxfeldt, 2015 | 2 | 0 | 1 |
| Martínez-García, 2015 | 2 | 0 | 1 |
| Huang, 2016 | 2 | 0 | 1 |
| Dalmases , 2015 | 2 | 0 | 1 |
| Woodson, 2014 | 2 | 0 | 0 |
| Neikrug, 2014 | 2 | 0 | 0 |
| Lloberes, 2014 | 2 | 0 | 1 |
| Gottlieb, 2014 | 2 | 0 | 1 |
| Chasens, 2014 | 2 | 2 | 0 |
| Berry, 2014 | 2 | 0 | 0 |
| Schutz, 2014 | 2 | 0 | 0 |
| Phillips, 2013 | 2 | 0 | 1 |
| Pedrosa, 2013 | 2 | 0 | 1 |
| Martínez-García, 2013 | 2 | 0 | 1 |
| Diaferia, 2013 | 2 | 0 | 0 |
| Andrén, 2013 | 2 | 2 | 0 |
| Sivam, 2012 | 2 | 2 | 0 |
| Lee, 2012 | 2 | 2 | 1 |
| Kushida, 2012 | 2 | 2 | 1 |
| Hoyos, 2012 | 2 | 2 | 1 |
| Sharma, 2011 | 2 | 2 | 1 |
| Ryan, 2011 | 2 | 0 | 1 |
| Philips, 2011 | 2 | 0 | 1 |
| Kohler, 2011 | 2 | 0 | 0 |
| Drager, 2011 | 2 | 0 | 0 |
| Aarab, 2011 | 2 | 0 | 0 |
| Nguyen, 2010 | 2 | 2 | 0 |
| Lozano, 2010 | 2 | 0 | 1 |
| Lam, 2010 | 2 | 2 | 0 |
| Durán-Cantolla, 2010 | 2 | 2 | 1 |
| Barbe´, 2010 | 2 | 0 | 1 |
| Galetke, 2009 | 2 | 0 | 0 |
| Gagnadoux, 2009 | 2 | 0 | 1 |
| Damjanovic, 2009 | 2 | 0 | 0 |
| Siccoli, 2008 | 2 | 0 | 1 |
| Ruttanaumpawan, 2008 | 2 | 0 | 0 |
| Petri, 2008 | 2 | 2 | 1 |
| Kohler, 2008 | 2 | 2 | 1 |
| Hoekema, 2008 | 2 | 0 | 0 |
| Galetke, 2008 | 2 | 0 | 0 |
| Egea, 2008 | 2 | 0 | 1 |
| Cross, 2008 | 2 | 2 | 0 |
| West, 2007 | 2 | 2 | 1 |
| Smith, 2007 | 2 | 2 | 1 |
| Patruno, 2007 | 2 | 0 | 0 |
| Martinez-Garcia, 2007 | 2 | 0 | 0 |
| Lam, 2007 | 2 | 0 | 0 |
| Haensel, 2007 | 2 | 0 | 0 |
| Fietze, 2007 | 2 | 0 | 0 |
| Drager, 2007 | 2 | 2 | 0 |
| Coughlin, 2007 | 2 | 2 | 0 |
| Robinson, 2006 | 2 | 0 | 0 |
| Hui, 2006 | 2 | 0 | 0 |
| Campos-Rodriguez, 2006 | 2 | 2 | 0 |
| Usui, 2005 | 2 | 0 | 0 |
| Marshall, 2005 | 2 | 2 | 0 |
| Blanco, 2005 | 2 | 0 | 0 |
| Arias, 2005 | 2 | 2 | 0 |
| Masa, 2004 | 2 | 0 | 1 |
| Mansfield, 2004 | 2 | 0 | 6 |
| Lloberes, 2004 | 1 | 0 | 0 |
| Ip, 2004 | 2 | 0 | 0 |
| Hussain, 2004 | 2 | 0 | 0 |
| Gotsopoulos, 2004 | 2 | 2 | 0 |
| Barnes, 2004 | 2 | 0 | 0 |
| Woodson, 2003 | 2 | 2 | 1 |
| Kaneko, 2003 | 2 | 0 | 0 |
| Becker, 2003 | 2 | 0 | 1 |
| Tan, 2002 | 2 | 0 | 0 |
| Randerath, 2002 | 2 | 0 | 0 |
| Pepperell, 2002 | 2 | 2 | 1 |
| Gotsopoulos, 2002 | 2 | 2 | 0 |
| Monasterio, 2001 | 2 | 1 | 0 |
| Bardwell, 2001 | 2 | 1 | 0 |
| Barbe´, 2001 | 2 | 1 | 0 |
| Ballester, 1999 | 2 | 1 | 0 |
| Redline, 1998 | 2 | 1 | 1 |
| Ferguson, 1997 | 2 | 1 | 0 |
| Meurice, 1996 | 2 | 2 | 0 |
| Ferguson, 1996 | 2 | 0 | 0 |
