# Supplementary material for: Verifying the Relative Efficacy between Continuous Positive Airway Pressure Therapy and Its Alternatives for Obstructive Sleep Apnea: A Network Meta-analysis
Source: Front Neurol. 2017 Jun 28;8:289. doi: 10.3389/fneur.2017.00289 (PMC5487413; doi:10.3389/fneur.2017.00289)
Supplement: Supplementary file 2 [file Table_2.DOCX]

**Table S2. Searching strategy**

| **Source** | **PubMed** |
| --- | --- |
| **Disease** | "Sleep Apnea, Obstructive"[Mesh] OR Obstructive Sleep Apneas[tiab] OR Obstructive Sleep Apnea Syndrome[tiab] OR Obstructive Sleep Apnea[tiab] OR OSAHS[tiab] OR OSAS[tiab] OR Upper Airway Resistance Sleep Apnea Syndrome[tiab] OR Obesity-Hypoventilation Syndrome[tiab] OR Obesity Hypoventilation Syndrome[tiab] OR nocturnal apnea[tiab] OR nocturnal apnoea[tiab] OR obstructive sleep apnea hypopnea syndrome[tiab] OR obstructive sleep apnoea[tiab] OR obstructive sleep apnoea hypopnoea syndrome[tiab] OR obstructive sleep apnoea syndrome[tiab] OR sleep apnoea[tiab] OR sleep apnoea syndrome[tiab] OR sleep apnoea syndromes[tiab] |
| **Therapy** | "Physical Therapy Modalities"[Mesh] OR Physical Therapy Modality[tiab] OR Physiotherapy[tiab] OR Physiotherapies[tiab] OR Physical Therapy Techniques[tiab] OR Physical Therapy Technique[tiab] OR Neurological Physiotherapy[tiab] OR Neurophysiotherapy[tiab] |
| **Study Type** | "randomized controlled trial"[pt] OR "controlled clinical trial"[pt] OR "randomized controlled trials as topic"[mh] OR "clinical trials as topic"[mh] OR "controlled clinical trials as topic"[mh] OR placebos[mh] OR "random allocation"[mh] OR “double-blind method"[mh] OR randomized[tiab] OR placebo[tiab] OR randomization[tiab] OR randomly allocated[tiab] OR ((double[tw] OR treble[tw] OR triple[tw]) AND (mask* [tw] OR blind* [tw])) |
|  |  |
| **Source** | **Embase** |
| **Disease** | 'sleep disordered breathing'/exp OR 'Obstructive Sleep Apneas':ab,ti OR 'Obstructive Sleep Apnea Syndrome':ab,ti OR 'Obstructive Sleep Apnea':ab,ti OR 'OSAHS':ab,ti OR 'OSAS':ab,ti OR 'Upper Airway Resistance Sleep Apnea Syndrome':ab,ti OR 'Obesity-Hypoventilation Syndrome':ab,ti OR 'Obesity Hypoventilation Syndrome':ab,ti OR 'nocturnal apnea':ab,ti OR 'nocturnal apnoea':ab,ti OR 'obstructive sleep apnea hypopnea syndrome':ab,ti OR 'obstructive sleep apnoea':ab,ti OR 'obstructive sleep apnoea hypopnoea syndrome':ab,ti OR 'obstructive sleep apnoea syndrome':ab,ti OR 'sleep apnoea':ab,ti OR 'sleep apnoea syndrome':ab,ti OR 'sleep apnoea syndromes':ab,ti |
| **Therapy** | 'physiotherapy'/exp OR 'Physical Therapy Modality':ab,ti OR 'Physiotherapy':ab,ti OR 'Physiotherapies':ab,ti OR 'Physical Therapy Techniques':ab,ti OR 'Physical Therapy Technique':ab,ti OR 'Neurological Physiotherapy':ab,ti OR 'Neurophysiotherapy':ab,ti |
| **Study Type** | random*:ab,ti OR placebo*:ab,ti OR 'double blind procedure'/exp OR 'triple blind procedure'/exp OR 'randomized controlled trial'/exp OR 'controlled clinical trial'/exp OR 'crossover procedure'/exp OR 'random allocation':ab,ti OR placebo:ab,ti OR 'randomized controlled trial':ab,ti OR 'randomised controlled trial':ab,ti OR rct:ab,ti OR 'randomly allocated':ab,ti OR 'allocated randomly':ab,ti OR ((double:ab,ti OR treble:ab,ti OR triple:ab,ti) AND (blind:ab,ti OR mask:ab,ti)) |
