# Supplementary figures and images for: Verifying the Relative Efficacy between Continuous Positive Airway Pressure Therapy and Its Alternatives for Obstructive Sleep Apnea: A Network Meta-analysis
Source: Front Neurol. 2017 Jun 28;8:289. doi: 10.3389/fneur.2017.00289 (PMC5487413; doi:10.3389/fneur.2017.00289)

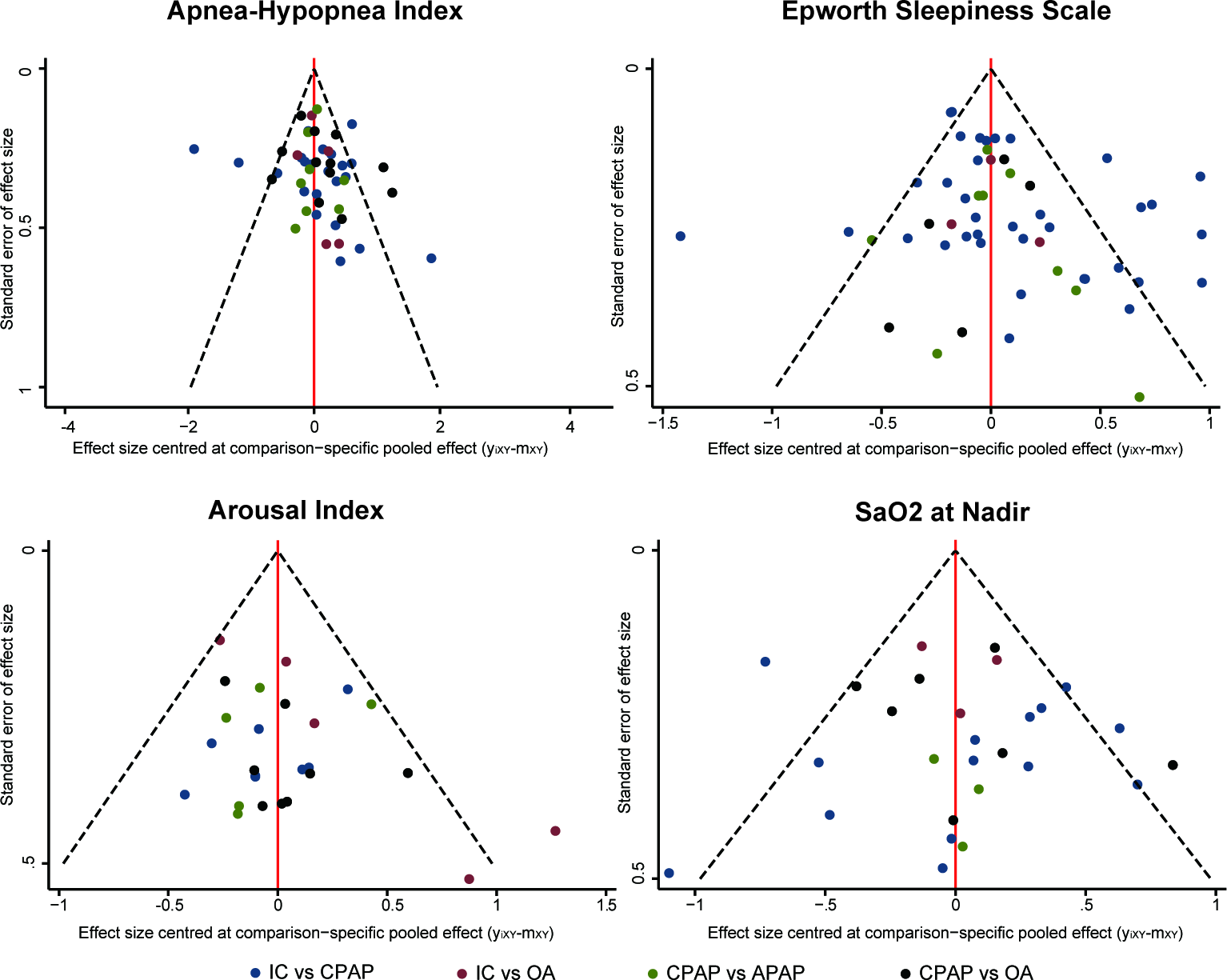

Supplement: Supplementary file 3 [file Image_1.TIF]

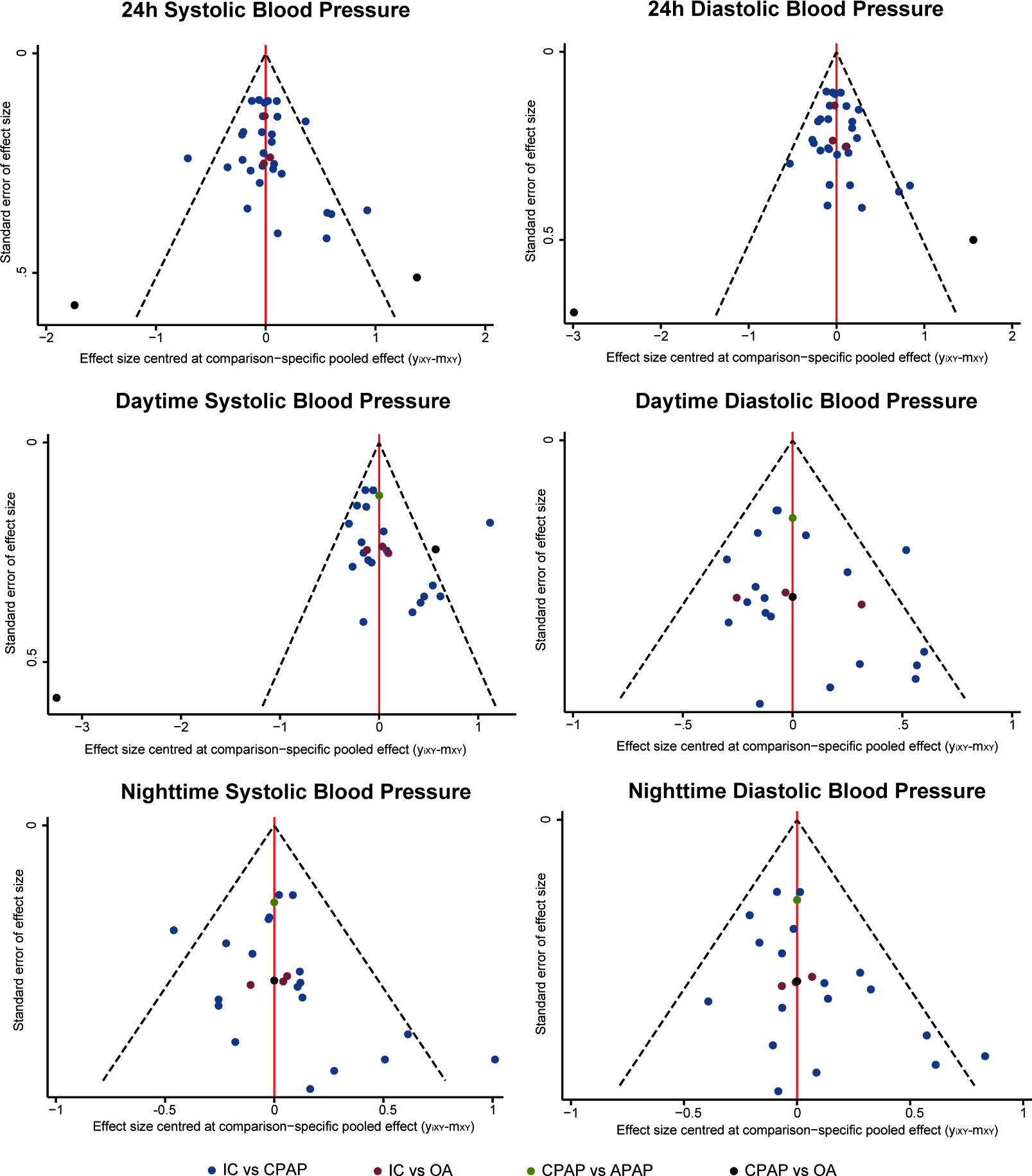

Supplement: Supplementary file 4 [file Image_2.TIF]

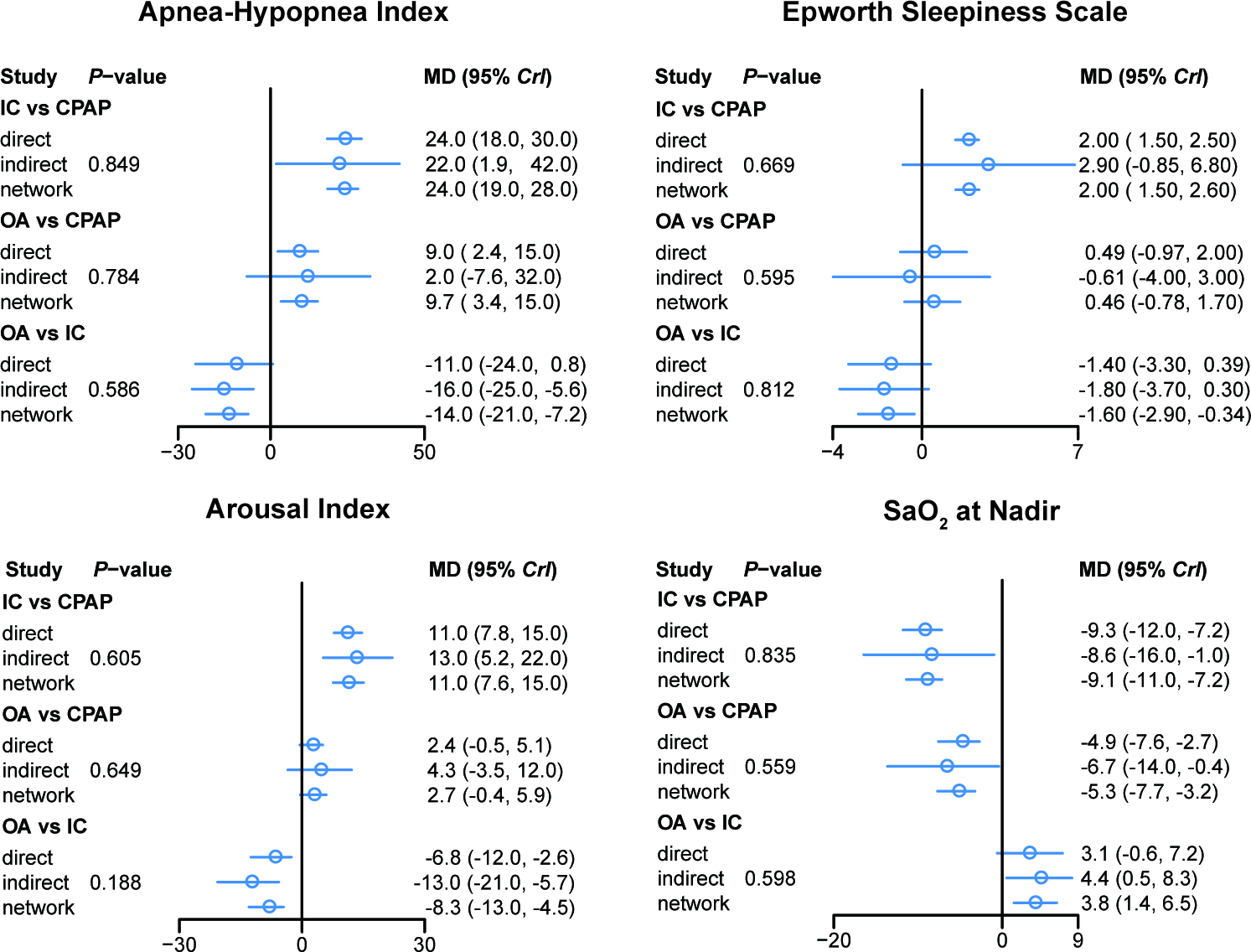

Supplement: Supplementary file 5 [file Image_3.TIF]

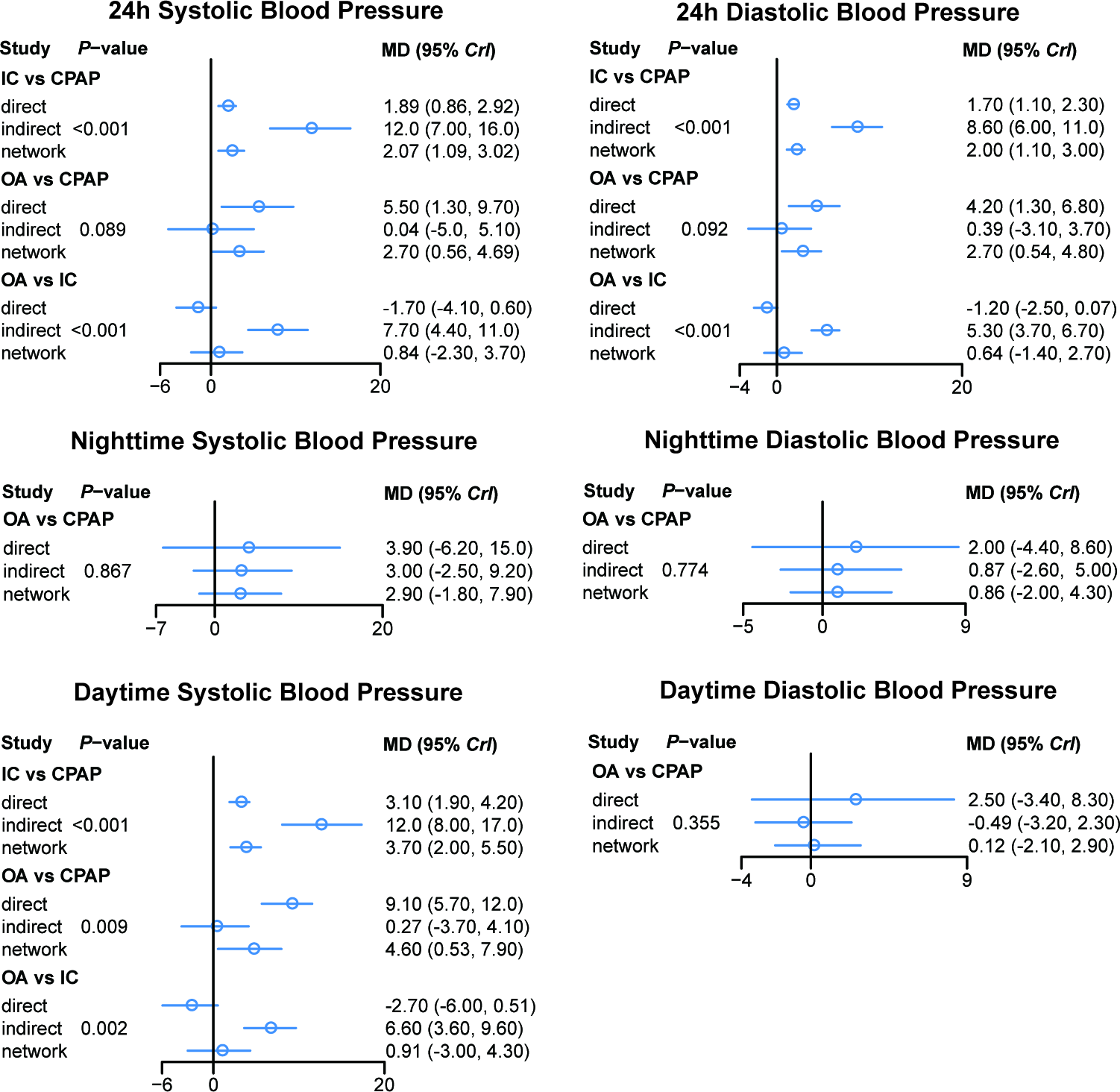

Supplement: Supplementary file 6 [file Image_4.TIF]

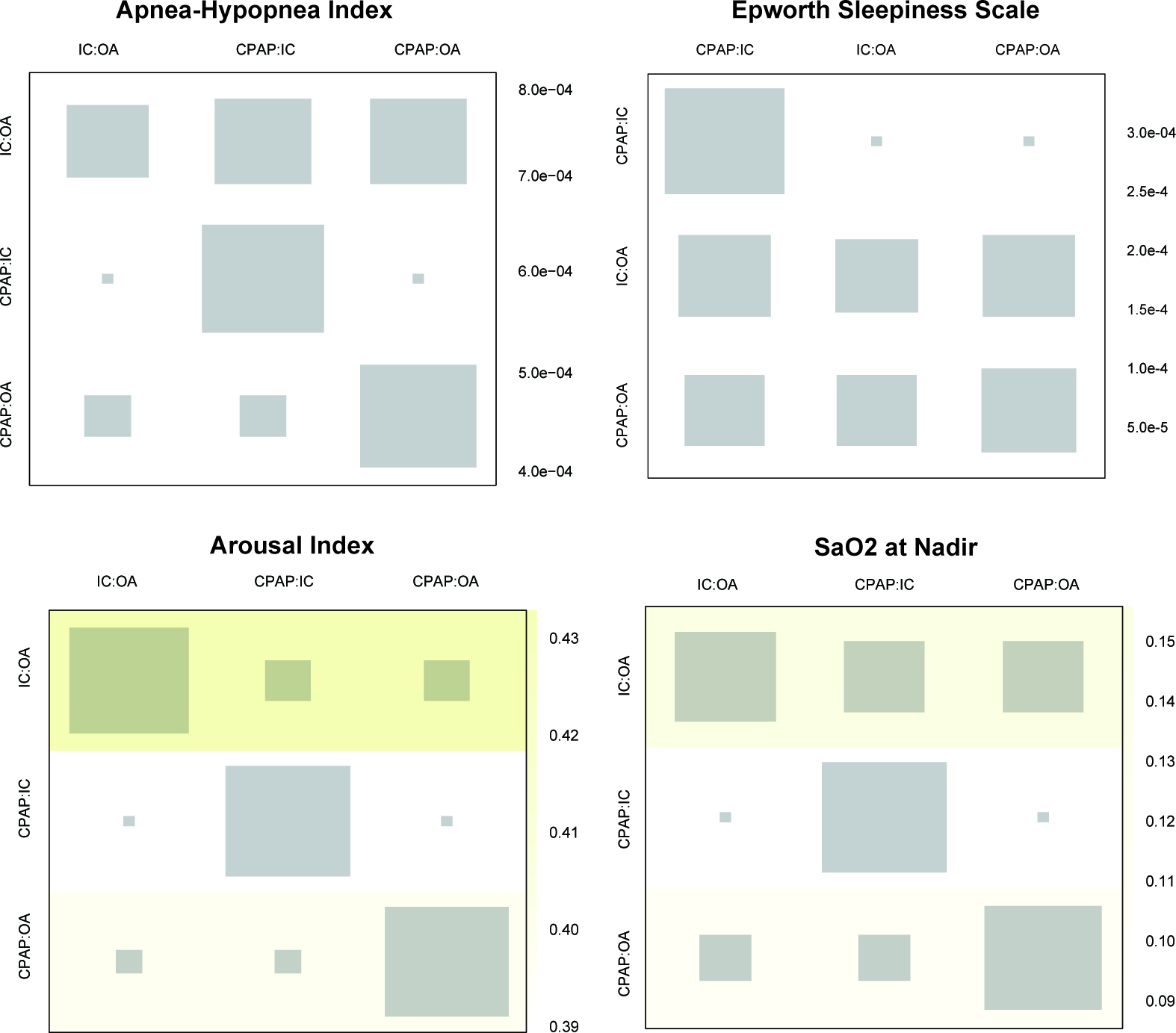

Supplement: Supplementary file 7 [file Image_5.TIF]

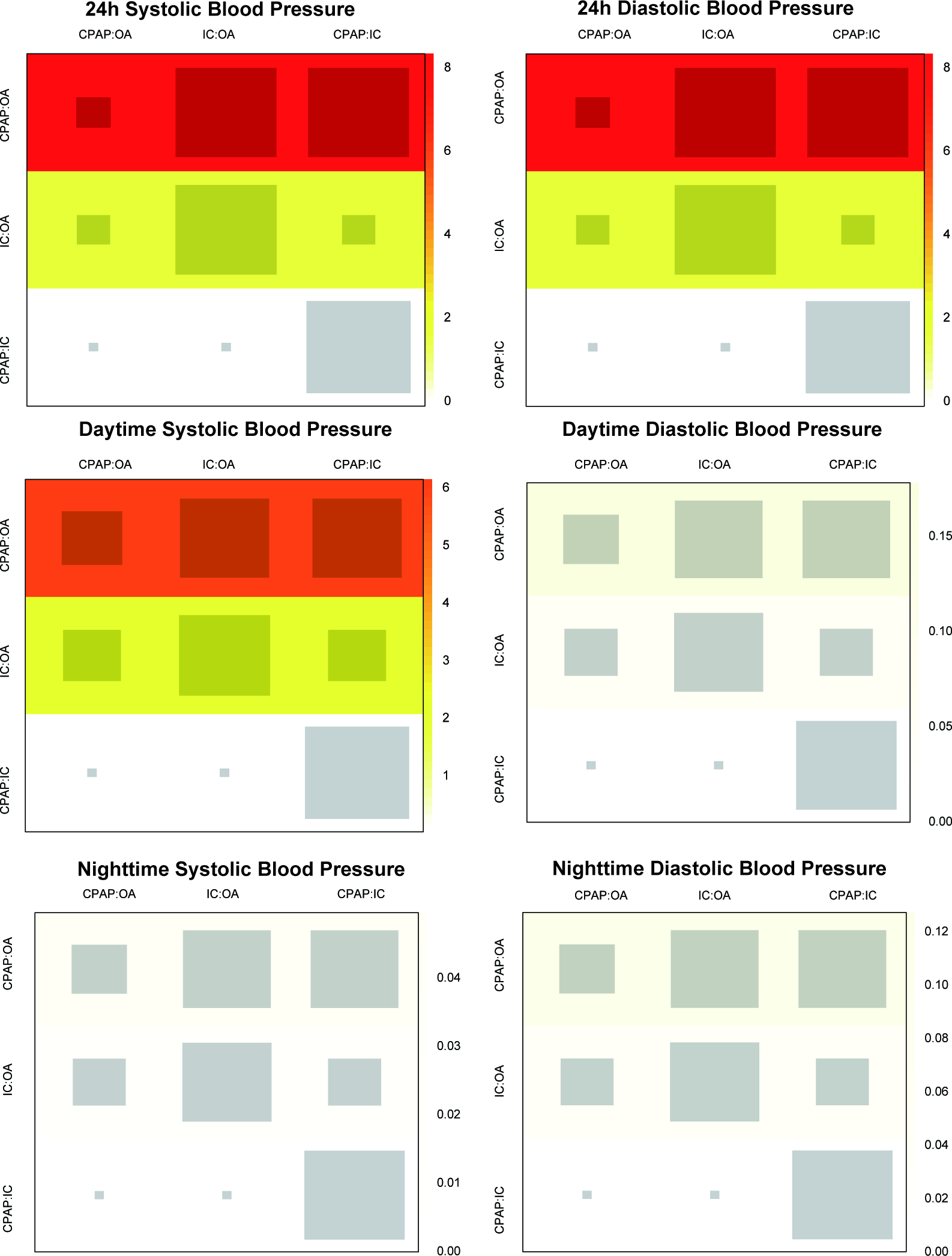

Supplement: Supplementary file 8 [file Image_6.TIF]
